# On the Combined Effect of Both the Reinforcement and a Waste Based Interfacial Modifier on the Matrix Glass Transition in iPP/a-PP-*p*PBMA/Mica Composites

**DOI:** 10.3390/polym12112606

**Published:** 2020-11-06

**Authors:** Jesús María García Martínez, Emilia P. Collar

**Affiliations:** Polymer Engineering Group (GIP), Polymer Science and Technology Institute (ICTP), Spanish Council for Scientific Research (CSIC), C/Juan de la Cierva, 3, 28006 Madrid, Spain

**Keywords:** organic–inorganic hybrid materials, compatibilizers, composites, modeling, interfaces, wastes, residues, iPP, aPP

## Abstract

This work deals with the changes of the glass transition temperature (T_g_) of the polymer in polypropylene/mica composites due to the combined and synergistic effect of the reinforcement and the interfacial modifier. In our case, we studied the effect on T_g_ of platy mica and an interfacial modifier with *p*-phenylen-bis-maleamic acid (*p*PBMA) grafted groups onto atactic polypropylene (aPP-*p*PBMA). This one contains 5.0 × 10^−4^ g·mol^−1^ (15% *w/w*) grafted *p*PBMA and was previously obtained by the author’s labs by using industrial polymerization wastes (aPP). The objective of the article must be perceived as two-fold. On one hand, the determination of the changes in the glass transition temperature of the isotactic polypropylene phase (iPP) due to both the reinforcement and the agent as determined form the damp factor in DMA analysis. On the other hand, forecasting the variation of this parameter (T_g_) as a function of both the interfacial agent and reinforcement content. For such purposes, and by assuming the complex character of the iPP/aPP-*p*PBMA/Mica system, wherein interaction between the components will define the final behaviour, a Box–Wilson experimental design considering the amount of mica particles and of interface agent as the independent variables, and the T_g_ as the dependent one, has been used. By taking in mind that the glass transition is a design threshold for the ultimate properties of parts based in this type of organic–inorganic hybrid materials, the final purpose of the work is the prediction and interpretation of the effect of both variables on this key parameter.

## 1. Introduction

The International Union of Pure and Applied Chemistry (IUPAC) defines nano-composite as that composite in which at least one of the phase domains has at least one dimension of the order of nanometers [1]. Furthermore, it defines a hybrid material as the one composed of an intimate mixture of inorganic components, organic components, or both types of components that usually interpenetrate on scales of less than 1 μm [1]. A detailed and comprehensive description and recommendations for studying this kind of system can found elsewhere [2]. The above-mentioned definitions match the iPP/mica composites, wherein the mica reinforcement has one dimension (thickness) in the nano-scale order close to 30 nm even if non-exfoliated [3,4]. In the same way, organic–inorganic hybrid material. In the same way, organic–inorganic materials can be revealed as multi-component compounds having at least one of their organic (the polymer) or inorganic component in the sub-micrometric and more usually in the nano-metric size domain [2,5]. It is worth it to mention that mica is a well-recognized mineral by its virtually perfect cleavage capacity at the atomic range level. It explains the excellent dimensional stability of mica/polymer composites in addition to excellent surface quality. Decidedly, the high-energy dissipation capabilities of mica confer the material a significant vibration and sound suppression. This fact makes it useful in many industries such as welding electrodes manufacture, gypsum plasterboards, paints, and rubber and plastics compounds [3,6]. Under these premises, the use of mica as the inorganic phase represents a wonderfully attractive way for obtaining iPP based organic–inorganic hybrid materials due to the fact that this mineral can be easily cleaved and delaminated in ultra-thin flakes with very high aspect ratios, making easy the alignment of the flakes in the matrix during the processing operations, providing high reinforcement level [6]. 

It is also completely accepted that organic–inorganic hybrid systems can be classified into two classes named Class I and Class II [5]. Class I organic–inorganic hybrid materials implies that the interaction between the phases is weak and principally due to Van der Waals, hydrogen, electrostatic, and so on, interactions [2,5]. On the contrary, Class II hybrids imply that the interaction between phases is intense due to real chemical bonds between them. The coexistence of both types of interactions (I and II) in the same system is also possible [2,5]. 

The above-mentioned atomic cleavage capacity represents a feature that confers almost total hydrophobicity to the mica mineral surfaces. The latter prevents the adverse effect at the mineral/matrix interface by the atmosphere water typically anchored to other silicate-based minerals. It explains the high dissipation capabilities below the glass transition temperature of the matrix in polypropylene/mica composites once the percolation threshold between the mica particles is reached [3,6]. Regardless, the affinity lack between the non-polar matrix and the polar mica particles still remains. Therefore, the employment of interfacial agents (or compatibilizers) has demonstrated an ability to enhance the interactions between the components in heterogeneous hybrid materials wherein one of the phases is a polymer [7,8,9,10,11,12,13]. Thus, it becomes desirable that the interfacial agent must resemble chemically the polymer phase besides demonstrating an affinity with the reinforcement. That means that in absence of an interfacial agent (due to the fact that mica and polypropylene exhibit very different polarity), the interface interaction level is relatively poor, and so, the inter-phase between components is weak. Consequently, the composite becomes a Class I organic–inorganic hybrid material. At this point, in spite of the research efforts performed, there is still much work to do for enhancing and precisely interpreting the complex phenomena taking place [2,3,4,11,12,13,14,15,16].

It is well worth it to establish that the remoter properties of the hybrid material strongly depend on the type and amount of interfacial agent used [7,11,12,13,14,15]. Besides, it is worth it to mention that usually just a scant amount of the interfacial agent is enough and mandatory to optimize the whole behavior of the hybrid material. Therefore, a critical amount of it jointly with those of the other components must emerge [3,4,7,8,15]. In addition, the authors reported the high dissipation capability of the matrix (iPP) below its glass transition when the percolation threshold between the mica particles is reached. The latter was performed for both injected and compressed composites by using DMA spectroscopy [3,8,13]. The iPP/aPP-*p*PBMA/Mica has been already characterized in previous works by tensile, flexural, and impact properties. Additionally, the crystalline content was determined by DSC, the mica content by TGA, and the distribution and orientation of the mica platelets imposed by the injection molding process used by SEM and FESEM [3,4,17,18,19,20].

Aditionally, the preferential location of whatever interfacial agent in the inter-phase between the reinforcement (mica) and the matrix (iPP) was first proposed from DMA results [3,4,6,8,12,13,17,18,19] and further confirmed by SIRM (Synchrotron Infra-Red Microscopy) [20]. Preliminary studies allowed postulating the existence of chemical bonds between the mica and the interfacial agent [20]. This fact suggests that the iPP/aPP-*p*PBMA/mica system may be classified as a class II hybrid material (or at least as a mixture of I and II) wherein strong interactions occur [5].

Notwithstanding, the production of this kind of organic–inorganic hybrid materials implies a complex scenario depending on processing, composition, functionality, and emerging morphology. This permited the researcher to obtain complex systems with consummate mastery at the different scales [3,4,5]. Therefore, a straightforward idea about what a complex system means consists of considering it as the one with many blocks able of exchanging stimuli between them and the surroundings depending on the contour conditions [3,4,21,22]. The former gives rise to behavior far from those expected from just the properties of the specific characteristics of the blocks (here iPP, mica, and aPP-*p*PBMA) that does not even give a glimpse of the behavior of the system itself [3,4,5,21,22]. Put differently, the complex systems require interaction effects between the blocks that overflow the expected by considering any additive effect of each of them [21,22]. In fact, these types of systems can be modeled by the so-called “agent-based models” [21,22]. Hence, the Box–Wilson surface response methodology for predicting the variation of the T_g_ with the combined effect of the mica and the interfacial agent content resembles these “agent-based models.” Therefore, this permits the interpretation of the effect of both variables in the T_g_ variation with a physical sense [3,4,23,24]. In fact, the Box–Wilson response surface methodology can be considered as an “Agent-based Model” since it considers blocks (here named controlled factors), and a series of interaction terms helping to detect other effects of the materials rather than those of the controlled factors (mica and aPP-*p*PBMA). In our case, the external stimuli remained minimized since the processing method is the same for all the samples [3,4,23,24]. Moreover, the possible emergence of optimal coordinates in the experimental space scanned let discriminate the efficient and the non-efficient components Therefore, this article concerns the study and prediction of the glass transition temperature (T_g_) looking to identify and interpret the combined and synergistic effect of the mineral reinforcement (mica), and the interfacial agent (aPP-*p*PBMA) on the variation and variability of the glass transition temperature of the polymer phase in the hybrid material as determined by Dynamic Mechanical Analysis [25,26].

## 2. Materials and Methods

### 2.1. Materials

**Figure 1:**
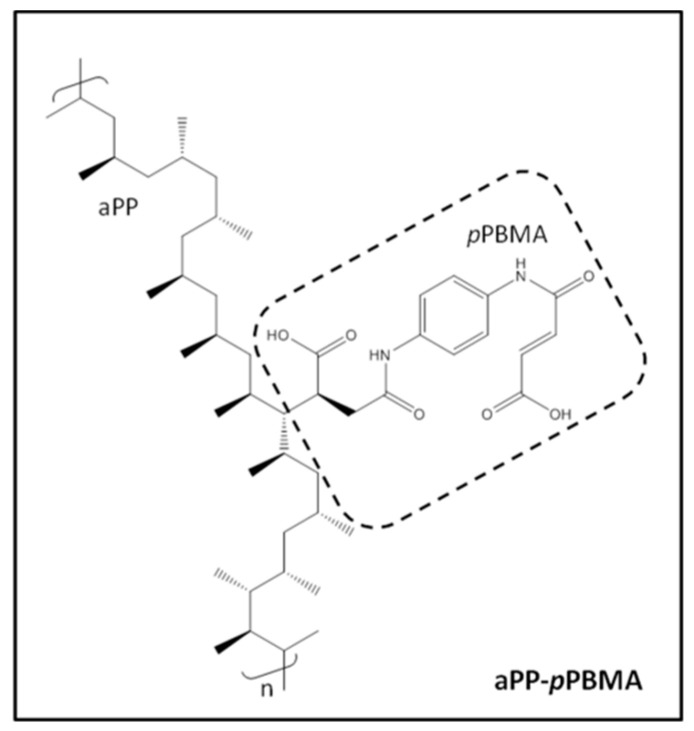
Chemical structure of the aPP-*p*PBMA interfacial agent used in this work.

The starting materials used were an isotactic polypropylene, iPP (ISPLEN 050) by Repsol (Madrid, Spain) with the following properties: ρ = 0.90 g/cm^3^; M_w_ = 334,400; M_n_ = 59,500; T_g_ = −13 °C, and phlogopite mica platelets (KMg_3_[Si_3_AlO_10_](OH)_2_), Alsibronz^®^ by BASF (Barcelona, Spain). The platy mica with density = 2.85 g/cm^3^; specific surface BET = 1.5 m^2^/g; average particle larger size = 79.8 µm was chosen by its demonstrated dimension stability, mean size and particle size distribution after and before the processing operations [3,4,14,17,18,20]. As interfacial agent, a grafted atactic polypropylene with 15% *w/w* (5 × 10^−4^ mol/g _polymer_) *p*-phenylen-bis-maleamic acid attached groups (aPP-*p*PBMA) designed and obtained by the authors through a chemical modification process in the melt by using polymerization wastes as raw material. A fully detailed description of the process and the characterization procedures of the grafted polymer are fully described elsewhere [18]. Figure 1 shows a scheme of the chemical structure of the interfacial agent used.

### 2.2. Sample Preparation

The composites, according the doses conditions in Table 1, were compounded in a Rheomix 600 chamber connected to a Rheocord 90 (Haake, Barcelona, Spain) by the at a time addition of the platy mica and the interfacial agent (aPP-*p*PBMA) to the previously the molten iPP (190 °C). The interfacial agent (aPP-*p*PBMA) was incorporated to the iPP by just replacing the same amount of it in the compound. Therefore, once the torque was stabilized, after five minutes blending, the chamber was opened and the composites were cooled down into and ice bath.

After that, once dried overnight at 25 °C, the hybrid material was milled to pellets and then injection molded at 200 °C in dog-bone type 1BA samples (ISO 527-2) by means of a Babyplast 6/6 micro-injection machine. From these, a series of prismatic samples (19.5 × 4 × 2 mm^3^) shaped according the DMA test requirements, were obtained.

The real particle content in the composite was determined by thermo-gravimetric analysis (TGA) and the particle distribution by Field Emission Scanning Electron Microscopy (FESEM) and Environmental Scanning Electron Microscopy (ESEM) in previous articles by the authors [3,4,7,13,17].

### 2.3. Characterization

We used a dynamic mechanical analyzer, DMA, (METTLER DMA861, Madrid, Spain) under the tension mode to obtain the DMA spectra of all the compounds in Table 1. For such purpose, we followed the recommendations of ASTM D5026 standards. In this way, the dynamic mechanical parameters were measured within the range of linear viscous-elastic behavior of the material by considering 12N oscillating dynamic force applied at a fixed frequency (1 Hz) and 3 μm amplitude being the heating rate equal to 2 °C/min. The temperature was varied in the −40 to 60 °C interval. We use this frequency for better determining the interfacial effects. Moreover, both the rather low frequency and displacement applied to the samples in the DMA are due to avoid whatever nonlinear behaviour and any morphological changes provoked by eventual internal heat generation.

### 2.4. Mathematical Model

We employed Box–Wilson statistical experimental design (sDOE) to study and predict the glass transition variation of the iPP/aPP-*p*PBMA/mica organic–inorganic hybrid system. In essence. this methodology is in a central rotary composite design conssiting in (2^k^ + 2k + 1) experiments augmented with (2 + k) replicated runs in the central point coded as (0,0). Here, k is the number of the independent variables chosen (in our case mica and aPP-*p*PBMA) [23,24]. Therefore, and in order to obtain samples in the desired ranges, 0% up to 40% in mica content and 0% up to 10% for aPP-*p*PBMA in the organic–inorganic hybrids, an interval between 14.4% and 35.6%, and 1.465% and 8.535%, respectively must be considered, the latter is just the consequence of the factorial component coded as (−1, 1) in Box–Wilson methodology for the experimental space to be studied. Equally, the coded variable for the star points of the model is α = √2 [23,24]. This coding, jointly with the uncoded (named as controlled) variables, have been included in Table 1. With this premise, the glass transition temperature measured for each experiment can be fitted, and thus a polynomial predicting (if adequate correlation is obtained) this property within the experimental range studied is obtained [23]. This information is included in Table 2 and Table 3.

## 3. Results and Discussion

**Figure 2:**
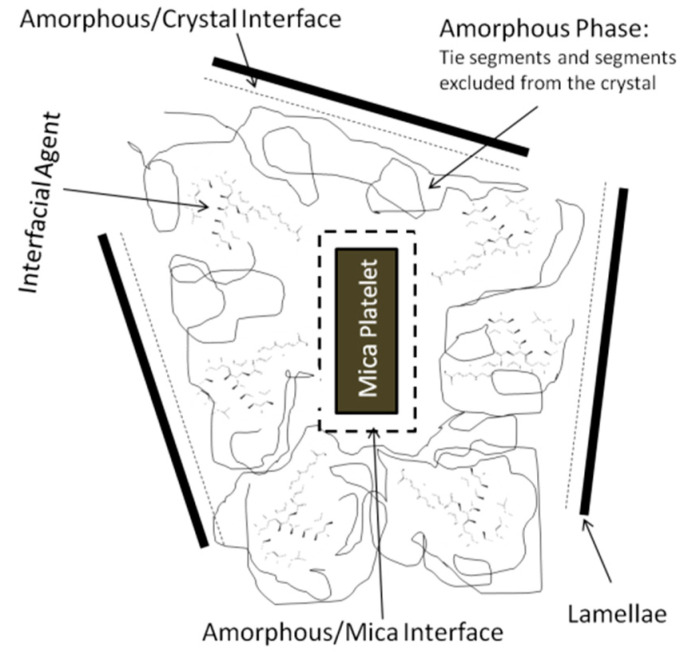
Scheme of the possible iPP/aPP-*p*PBMA/mica interactive scenario.

Prior to discussing the DMA results, it is worth briefly and properly describing the scenario and possible interactions occurring when a polymer matrix (iPP in our case) hosts platelet-like reinforcement (here, mica), and an interfacial modifier is also present (aPP-*p*PBMA). This model was previously underlined by the authors elsewhere [3]. In fact, in the discussion on the glass transition results as DMA measured, we follow the theoretical approach that describes the scheme in Figure 2. This one primarily considers two diverse types of amorphous fraction in the iPP matrix [3]. One of them would be that allocated between the mica particle and the closest iPP crystalline phase surrounding it, while the other would be that interconnecting the crystals within the iPP crystalline macroaggregates. The latter ensures the matrix continuity as a whole and chiefly determines the iPP relaxation behavior well above the glass transition region. This is because it is primarily related to the processing and molding conditions. At the effects of the present discussion, it is the former that mainly influences the glass transition range. Regardless, it depends on the overall so-called “free amorphous” fraction within the net amorphous PP amount. This is mainly commanded (in these hybrid materials) by that fraction’s highly constrained involvment in imbibing the inorganic particles.

To begin, we can observe a simplification of the complex scenario related to the interactions occurring at the organic–inorganic interface. Initially, we appreciate that between the iPP crystalline phase (referred to as lamellae) and the inorganic phase, a series of zones can be defined. The first one between the crystal and the amorphous phase of the iPP matrix. Here, we can identify an amorphous/crystal interphase wherein the amorphous (not ordered) sequences of the isotactic polypropylene may be allocated jointly with some atactic sequences of iPP. Additionally, the inorganic phase (mica) is mandatorily embedded in the amorphous phase [3,4,6,7], and so, an amorphous/mica interface can be defined. Additionally, in between the iPP lamellae and the inorganic phase, the amorphous phase of the system, consisting of tie segments and other segments excluded from the crystal, is identified. Thus, and since the grafted groups are also excluded, from the crystalline domains, the interfacial agent, whatever its origin (isotactic or atactic) must be preferentially hosted in this area [3,4,7,8,11,12,17,18,19,20]. Even more, the interfacial agent used here (aPP-*p*PBMA), due to its amorphous origin, must be mandatorily allocated in this phase [3,4,7,8,12,13,17,18]. Hence, it results in clear that the presence of both the inorganic phase and the interfacial agent in the amorphous domains must disrupt this one. Consequently, it must influence the mobility of this amorphous phase responsible for the glass transition of the polymer. Thus, and depending on how crowded the zone is (by the presence of mica and aPP-*p*PBMA) and how intense the interactions are between them and the matrix, this transition must be necessarily affected [3,4,6,7].

In this sense, to replace a minor fraction of the polymer matrix with an interfacial agent greatly improves the interactions throughout the dynamic interface between the polymer and the reinforcement [3,4,5,8,12,13,17,18]. This implies the existence of a critical value depending on both the amount and the type of the interfacial agent used, and the processing history of the organic–inorganic hybrid material [7,17,18]. Notwithstanding, this is a core concept that is too often not considered in plenty of works in literature that do not pay attention to the processing and shaping operations. Therefore, these studies avoid considering that the properties of a polymer-based material strongly depend on how it has been conducted to the solid-state.

In a way, when studying the effect of the interfacial agent in an organic–inorganic hybrid composite consisting of changing the transport phenomena throughputs, we consider two possibilities. On one hand, the researcher can wield a constant amount of interface agent by varying the grafting level [11,12]. On the other, the interfacial agent may vary by keeping constant the graft percentage [3,4,7,17,18]. This last one represents the route we have adopted in the present study.

Additionally, the preferential orientation of the flow elements governing the preferential alignment of the platelets, their morphological variations, the particle size, and size distribution changes caused by the processing steps must not be assigned to modifications of the inter-phase (as many frequently occur in literature). In this sense, it is mandatory to indicate that the inorganic platelets used (mica) did not suffer significant changes in particle size and particle size distribution during processing [3,4,17,18,19,20]. This is the reason why a platy mica (KMg_3_[Si_3_AlO_10_](OH)_2_) providing a real reinforcement effect to the organic–inorganic material was chosen for this study [3,4,6,17,18,19,20]. Consequently, the real and precise mica content in the material must be ascertained. The latter in order to not falsely identify other interfacial effects than the interfacial agent and the mica caused by mere changes in the flow-dynamic of the system (and then hardly traceable). The latter was checked by the authors for the same experimental worksheet by means of TGA analysis [3,4,7,17,18].

### 3.1. Dynamic Mechanical Spectra: Determination of T_g_

**Figure 3:**
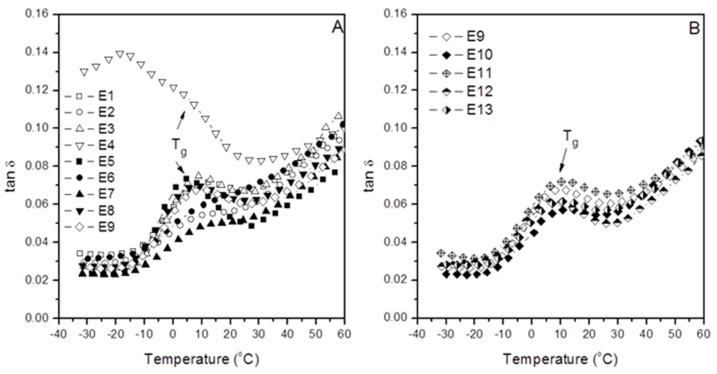
Evolution of the damp factor with temperature and glass transition for the indicated samples: (**A**) Central Rotary Composite Design Runs and (**B**) Central Point Replicated Runs.

Here, we discuss the glass transition variations observed by the loss factor (tan δ) as determined by DMA for the iPP/Mica organic–inorganic hybrid material and the way this parameter is influenced by the combined effect of both the inorganic phase (mica) and the interfacial modifier used (aPP-*p*PBMA). Therefore, Figure 3 shows the evolution of the tan δ with temperature for all the samples of the Box–Wilson worksheet in Table 1. In this work, we have merely used the values for the glass transition temperature obtained from the tan δ plots in Figure 3. Typically, in the case of an iPP based composite this transition appears between −10 and 40 °C [3,4,7,11,12,17,18,19], being in our case between 6.2 °C (sample E5) up to 10.9 (sample E7), depending on the hybrid material formula (Table 1). It is significant to mention that this transition is related to the cooperative chain segments’ motion on the “free” amorphous phase of the polymer wherein short-range diffusive chain motions takes place in spite of the low dissipation capability due to mere atomic vibration motions [3,4,7,11,12]. All the values of the glass transition temperature have been compiled in Table 1. Hence, as mentioned hitherto, a maximum difference of 4.6 °C between experiment E7 with 25% mica and a tiny amount of aPP-*p*PBMA ratio equal to 25/0; T_g_ = 10.9 °C and E5 with mica/aPP-*p*PBMA ratio equal to 10/5; T_g_ = 6.2 °C, suggesting that the combined effect of mica and aPP-*p*PBMA greatly affect the glass transition value. In the same sense, it is important to mention that in all the cases the T_g_ observed is higher to that of the neat iPP as determined by DMA under the same conditions (T_g_ = 4 °C) [2,3], and so we find a maximum difference between the neat iPP and the slightly modified compound (E7) of 6.9 °C, and a minimum of 2.2 °C if compared with the E5 sample, containing 10% of mica and 5% of aPP-*p*PBMA, suggesting that there is the combined effect of the reinforcement and the interfacial agent what affect the glass transition.

Figure 3 shows the evolution of the loss factor with the temperature of each one of the compounds in Table 1. At a glance, we observe that the sample E4, with a 35.6/8.5 mica/aPP-*p*PBMA ratio, reveals a different pattern than the others. Thus, the latter evidence the abrupt increase in the dissipation capabilities of the iPP matrix in this organic–inorganic compound, similar to the reported for the 75/25 iPP/mica unmodified compound elsewhere [2,3,11,12]. The latter was explained on the basis of the well-known flatness of the mica particles rendering much amorphous iPP to imbibe them [3,4,12,13]. Therefore, in our case, the amount of mica is much higher (35.5%) but the amount of the amorphous character aPP-*p*PBMA is high enough as to provide sufficient extra amorphous phase aiding to embed the mica particles. Therefore, we found a similar dissipation mechanism in the case of a 25% unmodified mica compound [3,4,12,13] as in a 35.5/8.35 mica/aPP-*p*PBMA composite. On the contrary, just a tiny amount of aPP-*p*PBMA (sample E5) is enough to amend the interactions and so to alter the pattern regarding the unmodified iPP/Mica 75/25 unmodified composite [3,4,12,13]. The latter informs about the remarkably complex scenario that emerged from the possible interactions modeled in Figure 1. The mica particles act to disrupt the polymer bulk and consequently oblige a fraction of the polymer segments to be ordered [3,4,12,13]. Consequently, the amorphous region trapped at the iPP/Mica interface (Figure 1), which is coating the mica particles, is abruptly constrained. As follows, just a minor portion of the amorphous phase can become mobile [3,4,8,12,13]. Conversely, the presence of aPP-*p*PBMA in these regions of the organic–inorganic hybrid material may play a two-fold effect. On one hand, since its presence introduces supplementary amorphous material to the system, making more mobile one of the iPP phases, a decrease in the glass transition may be expected. On the other, the interactions between the *p*PMBA groups would be on the contrary sense by interacting with the mica domains. Thus, a complex scenario having influence in the ultimate value of the glass transition values looks to emerge. In fact, from the data and results in Table 1, it is impossible to discriminate at a glance about the effect of the components of the hybrid material on the values for the T_g_. Under these auspices, the use of Box–Wilson methodology is revealed as a reliable tool to interpret the results.

### 3.2. Polynomial Fits and Analysis of Variance (ANOVA)

Table 1 compiled the values for T_g_ for the experimental design followed. The *w/w* amounts of mica and the interfacial agent (aPP-*p*PBMA), the controlled factors have been also listed. Thus, the T_g_ for each one the samples were fitted to a quadratic model by means of Box–Wilson surface response methodology [25] obtaining a polynomial describing the evolution of the glass transition temperature. Consequently, Table 2 compiles the terms of the polynomial obtained together to the lack of fit and the confidence factor coefficients for ANOVA (analysis of variance). Hence, we observe a value for equal to 91.56%, excellent for a quadratic model since values for this parameter higher than 75% are considered as good for these kinds of models [23,24]. Additionally, this Table 2 includes the “lack of fit” (LF), which is related to the percentage of pure error due to any factors overlooked by the model but significant enough in the final prediction of it. At this place, we obtain a value of LF equal to 5.1%, indicating that just this value may explain other factors ignored by the model. Likewise, the extraordinary value for the confidence factor (CF = 99.5%) indicates the accuracy and significance of the chosen independent variables chosen to model the T_g_ evolution of the iPP/aPP-*p*PBMA/Mica organic–inorganic hybrid material for the whole experimental space studied.

**Figure 4:**
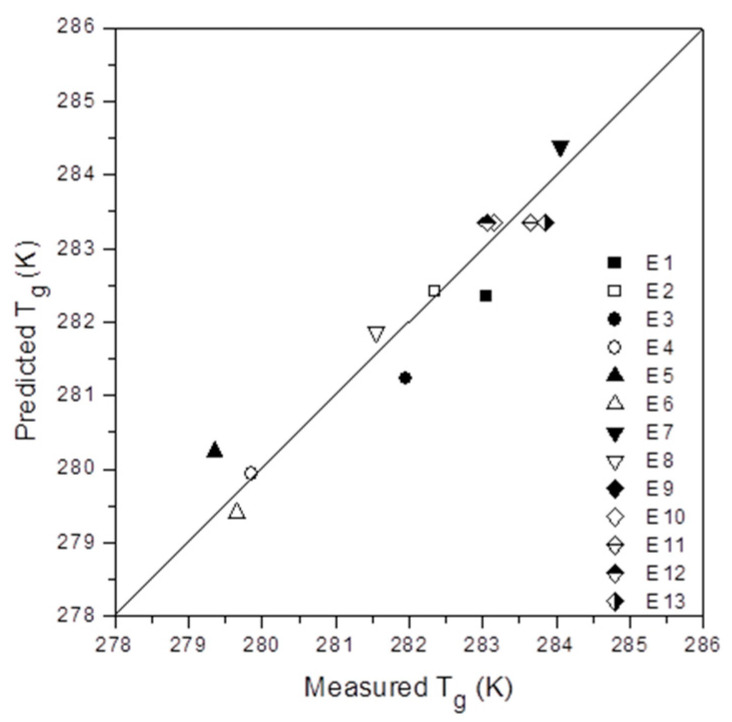
Measured versus predicted values for the glass transition temperature (T_g_).

Moreover, the parameters included in Table 2 robustly confirm the possibility of studying this organic–inorganic hybrid system by means of the Box–Wilson predictions. In any case, it is important to check the limitations of the model. For such a purpose, the Figure 4 shows the scatter plots for the predicted versus the measured glass transition temperature. Thus, we can observe the excellent correlation between them.

Hence, Table 3 compiles the confidence coefficient (%) and t-values for all the terms of the Box–Wilson polynomial relating the T_g_ with the composition of the hybrid material. At a glance, we notice the significance levels for each one of the parameters in the polynomial. Thus, by taking in mind that the more influence terms are those with t-values higher than two [23,24], we see that is the reinforcement that exercises more influence on the final T_g_ values; thus, the t-value = 7.0 for [Mica], and 7.2 for [Mica]^2^ or confidence factors of 99.9% for both of them. The latter agrees with the above-mentioned point about the disruption capability of mica in order to immobilize the amorphous phase participating in the glass transition phenomena. However, these values for the interfacial agent in isolation are small (0.25 and 0.48 for the linear and the quadratic term, respectively), indicating that the interfacial agent in isolation has little influence in the final value of the T_g_ of the iPP matrix. However, the combined effect of mica and aPP-*p*PBMA appears to influence the final values of T_g_ since the t-value for the interaction term is 1.12, indicating that the confidence coefficient is close to 70%. The latter suggests that the effect of the interfacial agent is important enough to modulate the change in T_g_ mainly depending on the ability of mica to disrupt the amorphous phase of the iPP matrix [3,4,5,12,13].

### 3.3. Influence of the Composite Composition in the T_g_ as Determined by DMA Spectra

As mentioned in the previous sections, and prior to discussing the model predictions, we must make a series of remarks about the experimental data compiled in Table 1. It is not the case that the more interface agent and mica contents, the more change in property, which appears to indicate that the behavior of the iPP/Mica system is highly complex, and the effect of the presence of the interfacial agent is not evident. The latter implies this aspect may hardly be ascertained with just classical random experiments. Furthermore, the existence of critical values in the component concentration derived from the interactions between them rather than the effect of the components in isolation is what determines the overall behavior of the iPP glass transition evolution, as has been demonstrated for other mechanical properties [7,17,18,19] and also by DMA [3,4]. Notwithstanding, the evolution of the glass transition temperature of the iPP phase (in this organic–inorganic hybrid material) with the amount of filler and interfacial modifier can be studied and discussed on the basis of the Box–Wilson model predictions.

**Figure 5:**
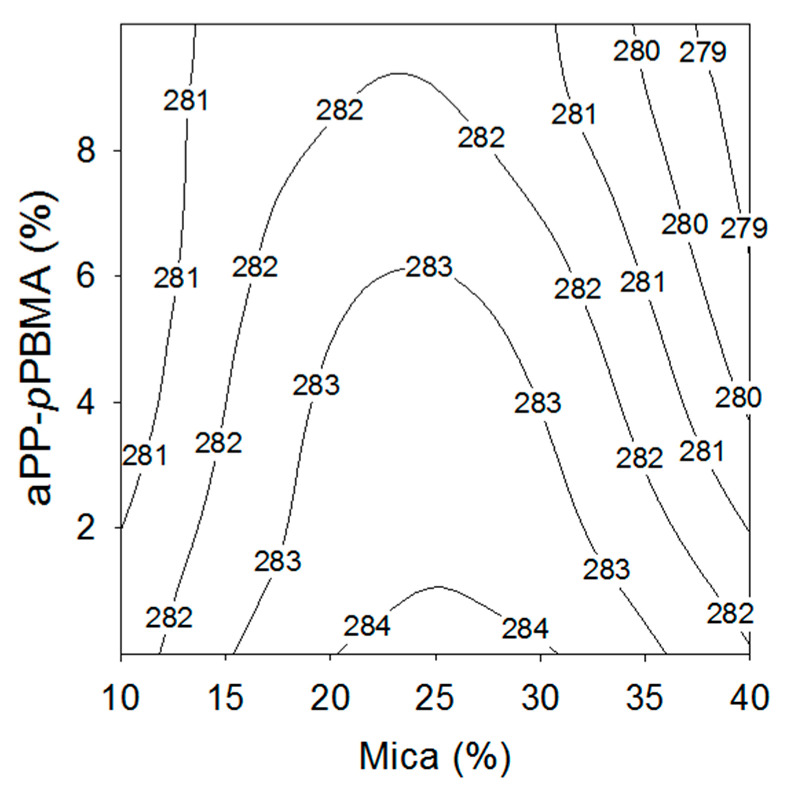
Evolution of T_g_ (K) with the Mica and aPP-*p*PBMA contents.

Thereinafter, Figure 5 shows the contour map of the glass transition of iPP as a function of the content of mica and interfacial agent. This map follows a rising ridge evolution, which means the existence of optimal coordinates in the experimental space scanned [23]. In our case, we observe quite close isolines for values below 15% and above 35% in mica content for whatever the amount of aPP-*p*PBMA, indicating there is a critical value for the mineral content, and the effect of increasing amounts of aPP-*p*PBMA is in the way to decrease the T_g_. In fact, between 15% and 35% of mica, the separation of the isolines increases implying that for such reinforcement concentration the influence of the interfacial agent is less evident. Notwithstanding, the existence of a critical point is firmly established.

**Figure 6:**
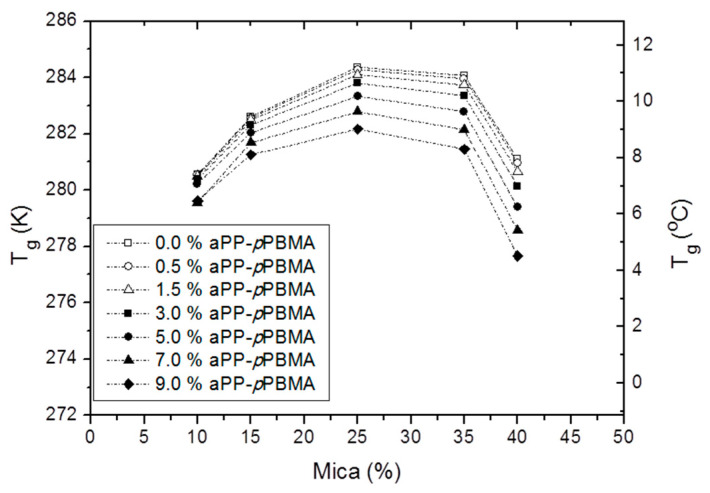
Evolution of T_g_ with the mica content at the indicated amount of interfacial agent.

Additionally, and in order to facilitate a better comprehension of the Box–Wilson predictions, we have included two parametric plots wherein the existence of critical points is once more evidenced. Therefore, in Figure 6 we have represented the T_g_ versus the mica content in the hybrid material for the indicated amounts of aPP-*p*PBMA. Although all the curves follow a similar pattern with a maximum variation of T_g_ for the compound with 25% of mica independently to the aPP-*p*PBMA content, we observe that in all the cases the effect of aPP-*p*PBMA decreases the glass transition of the iPP phase, indicating the amorphous phase of the interfacial agent cooperates in hosting the mineral particles, and so a portion of the amorphous phase of the iPP matrix now becomes free as to participate in the local motions governing such transition, and consequently, the glass transition may occur at a lower temperature. What is clear is that the critical point in the system is the 25% of mica, for whatever the quantity of the interfacial agent used, meaning that at this point the reorganization capabilities of the crystal/amorphous balance of the iPP are optimized, and the role of aPP-*p*PBMA implies slight modulation of this effect.

**Figure 7:**
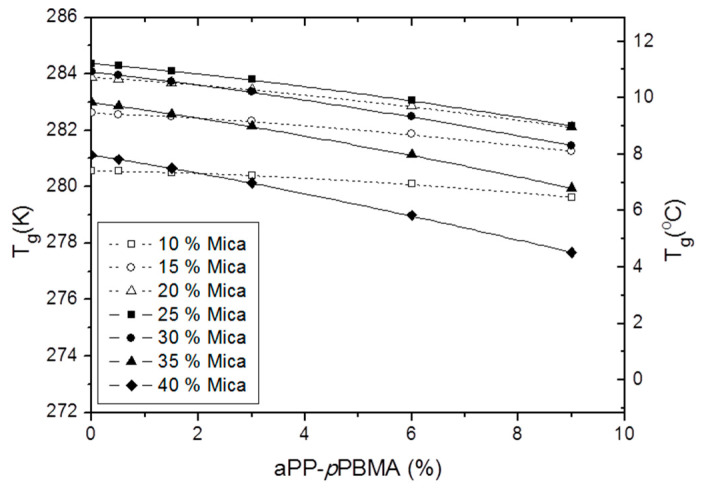
Evolution of T_g_ with the aPP-*p*PBMA content at the indicated amount of reinforcement.

In the same sense, Figure 7 plots the evolution of the glass transition of the iPP phase of the hybrid material with the aPP-*p*PBMA for different contents of mica. At a glance, we observe two families of curves with a similar slope to each other. One of the families, for values of mica below 25%, and other for values for mica 25% and higher. In this manner, the influence of the interfacial agent is in the way of decreasing the T_g_ of the iPP phase for whatever the amount of aPP-*p*PBMA used, but below 25% mica the decrease causes is low, due to the fact that the amount of amorphous phase of the iPP coating the mineral particles is not so constrained, and so the effect of the additional amorphous phase provided by the interfacial agent is not causing a considerable drop in the T_g_ values. However, values of mica equal to 25% and higher imply that this amorphous phase is therefore constrained that almost all of it is implied in covering the mineral and so, the difficulty of becoming mobile as to freely participate in the glass transition is high enough as to be sensitive to the incorporation of an additional amorphous phase coming from the interfacial agents aiding to coat the mineral flakes. At any rate, the more significant variations are found for the 25% mica compound, indicating that is the real critical point of the system, and so higher values than 25% imply lower T_g_ due to the different amorphous/crystal throughputs across the interphases.

To begin, the 25% mica coordinate is identified as the critical point of the system in the sense of increasing the glass transition value of the iPP phase. As follows, the interfacial agent plays a modulation effect on this transition. Nevertheless, the existence of this critical point maximizing this parameter is well evidenced. Furthermore, the fact that the latter is coincident to the determined for the ultimate properties (depending on the brittle to elastic capability) of the iPP/aPP-*p*PBMA/mica organic–inorganic hybrid material must be remarked [7,17,18,19].

## 4. Conclusions

The modulation role of aPP-*p*PBMA interfacial agent on the glass transition of the iPP phase of iPP/Mica composites has been determined by dynamic mechanical analysis (DMA). The use of the design of experiment methodologies (DOEs) has proved to be immensely useful in the study of systems wherein the interaction between the components determine the final property (the glass transition in this case). It is not the case that more of each component leads to a more outstanding level of interaction, which has been put into evidence once more. In fact, it is the existence of critical amounts of the components of the composite that governs the ultimate behavior of the system. In identifying the critical coordinates, the results obtained for the glass transition evolution agrees with those obtained for other ultimate properties. Therefore, this informs us of the linking of this threshold parameter (between the rigid-to-ductile behavior of the polymer matrix) with the ultimate behavior of the system. Thus, one emerging idea is that related to the effect of the reinforcement and the interfacial agent in the variation of the T_g_ of the polymers matrix. Here, we conclude that it depends on the interactions between the components in the sample jointly to the processing operations fingerprint. Consequently, those works in literature concluding that the effect on the T_g_ (increment or decrement of T_g_) caused by any component of the hybrid material in isolation adquire non-sense. This applies consistently if a well-defined and controlled scenario (in terms of composition and processing history) is not considered.

## Figures and Tables

**Table 1:** Experimental design and measured Glass Transition Temperature (T_g_) according to the Box–Wilson experimental worksheet.

	Controlled Factors *		Coded Factors			
Exp	x_1_ (%)	x_2_ (%)	x_1_	x_2_	T_g_ (°C)	T_g_ (K)
E1	14.4	1.465	−1	−1	9.9	283.05
E2	35.6	1.465	1	−1	9.2	282.35
E3	14.4	8.535	−1	1	8.8	281.95
E4	35.6	8.535	1	1	6.7	279.85
E5	10.0	5.000	−√2	0	6.2	279.35
E6	40.0	5.000	√2	0	6.5	279.65
E7	25.0	0.001	0	−√2	10.9	284.05
E8	25.0	9.999	0	√2	8.4	281.55
E9	25.0	5.000	0	0	9.9	283.05
E10	25.0	5.000	0	0	10.0	283.15
E11	25.0	5.000	0	0	10.5	283.65
E12	25.0	5.000	0	0	9.9	283.05
E13	25.0	5.000	0	0	10.7	283.85

* x_1_ = [Mica]; * x_2_ = [aPP-*p*PBMA].

**Table 2:** Statistical Parameters and Coefficients of the Polynomials. (Polynomial Equation: a_0_ + a_1_·x_1_ + a_2_·x_2_ + a_3_·x_1_·x_2_ + a_4_·x_1_^2^ + a_5_·x_2_^2^) *.

	<r^2^> (%)	LF (%)	CF (%)	Linear Terms			Interaction Term	Quadratic Terms	
				a_0_	a_1_	a_2_	a_3_	a_4_	a_5_
T_g_ [K]	91.56	5.1	99.5	274.1	0.8024	0.07167	−0.009341	−0.011567	−0.009048

* x_1_ = [Mica]; x_2_ [aPP-*p*PBMA].

**Table 3:** Confidence coefficient (%) and t-values for the different terms of model obtained for T_g_. (Polynomial Equation: a_0_ + a_1_·x_1_ + a_2_·x_2_ + a_3_·x_1_·x_2_ + a_4_·x_1_^2^ + a_5_·x_2_^2^) *.

	Linear Parameters		Interaction Parameter	Quadratic Parameters	
	x_1_	x_2_	x_1_·x_2_	x_1_^2^	x_2_^2^
T_g_[K]	7.0 (99.9%)	0.25 (25.6%)	1.12 (68.9%)	7.5 (99.9%)	0.48 (36.7%)

* x_1_ = [Mica]; x_2_ [aPP-*p*PBMA].
